# Impact of exercise intensity on oxidative stress and selected metabolic markers in young adults in Ghana

**DOI:** 10.1186/s13104-018-3758-y

**Published:** 2018-09-03

**Authors:** Patrick Diaba-Nuhoho, Emmanuel Kwaku Ofori, Henry Asare-Anane, Sylvester Yaw Oppong, Isaac Boamah, Dee Blackhurst

**Affiliations:** 1 Division of Vascular Endothelium and Microcirculation, Department of Medicine III, University of Technology Dresden, Fetscherstr. 74, 01307 Dresden, Germany; 2 Department of Physiology, University of Lausanne, Lausanne, Switzerland; 3 Department of Chemical Pathology, University of Ghana, Korle-Bu, Accra, Ghana; 4 Department of Microbiology, University of Ghana School of Medicine and Dentistry, Korle-Bu, Accra, Ghana; 5 Division of Chemical Pathology, University of Cape Town, Observatory, Cape Town, 7925 South Africa

**Keywords:** Antioxidants, Reactive oxygen species, Different intensity exercise

## Abstract

**Objective:**

This study aimed to evaluate the effect of different levels of exercise on markers of oxidative stress and selected metabolic parameters in Ghanaian young adults.

**Results:**

Significant increases in a marker of oxidative stress malondialdehyde and antioxidants such as superoxide dismutase and uric acid were observed in the exercisers compared with the inactive group (p < 0.05). Total cholesterol and high density lipoprotein levels were significantly different (p < 0.05) between the two groups. Positive associations between exercise intensity, antioxidant concentration and malondialdehyde were observed within the exercise group for vigorous exercise with regards to uric acid, superoxide dismutase and malondialdehyde (r = 0.512, p = 0.004; r = 0.810, p = 0.001; r = 0.715, p = 0.001) respectively and moderate exercise vs malondialdehyde (r = 0.841, p = 0.001) compared to the inactive group. Exercise participants performed more vigorous exercise (p < 0.001), moderate exercise (p < 0.001) and more walking (p < 0.001) compared with the inactive group while the inactive group exhibited more sitting (p < 0.001). The study provides a first report on the risk associated with increase in oxidative stress and the importance of walking as a health promotion intervention among young Ghanaian adults.

**Electronic supplementary material:**

The online version of this article (10.1186/s13104-018-3758-y) contains supplementary material, which is available to authorized users.

## Introduction

There is little doubt that physical exercise improves quality of life, reduces oxidative damage and prevents the incidence of several disorders including diabetes and cardiovascular disease [1–5]. Beneficial effects on the antioxidant system have also been reported [6, 7]. There is, however a continuous debate on exercise type, intensity, frequency and duration, on the health of an individual. Furthermore, research into the influence of exercise on the antioxidant system in a socio-cultural setting like Ghana remains largely unexplored. Ghana as a middle-income country has a rapidly-increasing urban society which is being increasingly exposed to technological environments that contribute to health changes [8, 9]. Though there is a drive to promote health worldwide through lifestyle changes, there is still limited knowledge of the amount of exercise performed or needed in the various population groups [10]. In addition, Ghana, like many developing countries, has limited data on exercise patterns of the different age groups. This is of importance when issuing public health recommendations [11]. A meta-analysis published from 15 studies between 1966 and 2007 estimates 13% of West African adults are physically inactive with low levels of physical activity and sedentary behaviour, and this may be associated with mental health problems in Ghana [12, 13]. A local study such as this might contribute to educating the local population, which in turn may reduce the burden of non-communicable diseases (NCDs) [14]. The aim of this study was to evaluate the effect of exercise on specific endogenous antioxidants and selected metabolic parameters in Ghanaian young adults.

## Main text

### Methods

#### Participant selection

The study was conducted between September 2014 and March 2015. Forty-four inactive subjects were age-matched with 42 regular exercisers. The exercisers were recruited from two gym centers (University of Ghana Sports Directorate Center and Yok 250 at Mamprobi) in Accra, Ghana, which had equipment such as Smith machines, shoulder and chest presses, adjustable cable crossovers, upright fitness bikes, a lap pull down and treadmills. Participants were considered exercisers if they undertook at least three formal exercises for 30 min per session at their gym centers each week. Volunteers who smoked, had any chronic illness (obesity, hypertension or diabetes), pregnant or lactating and those who consumed vitamins or antioxidants were excluded from the study. Subjects were considered inactive (controls) if they participated in less than 1 h of planned activity each week. A structured pre-tested interview questionnaire and exercise programme was administered to all consenting participants (see Additional files 1 and 2).

#### Anthropometric measurements

Height measurements were taken using a wall-mounted stadiometer (SEC-213, Birmingham, United Kingdom). Weight was measured in light clothing using a floor digital scale (Seca 770, Birmingham, United Kingdom). Body mass index (BMI) was computed as weight (kg)/height^2^ (m^2^). A body composition monitor (BC-533, Tanita innerscan™ United Kingdom) which employed bio-impedance analysis was used (see Additional file 3).

#### Laboratory procedure

Venous blood (5 mL) was obtained from the participants in the mornings between 07h00 and 09h00 each day after an overnight fast after participants had rested for 15 min, according to the Helsinki protocol declaration (2008) (see Additional file 3).

#### Data analysis

GraphPad prism software version 6.0 (San Diego California, USA) was used for data analysis. Values are expressed as mean ± standard deviation. Physical activity scores and levels were calculated using the compendium for physical activity and recorded as minutes/week [15]. Unpaired student *t*-test was used for the comparison between means, and post hoc Bonferroni’s test was used to evaluate differences within groups. Several associations were tested using Pearson’s correlation coefficient. Statistical significance was set at a p-value < 0.05.

### Results

**Fig. 1:**
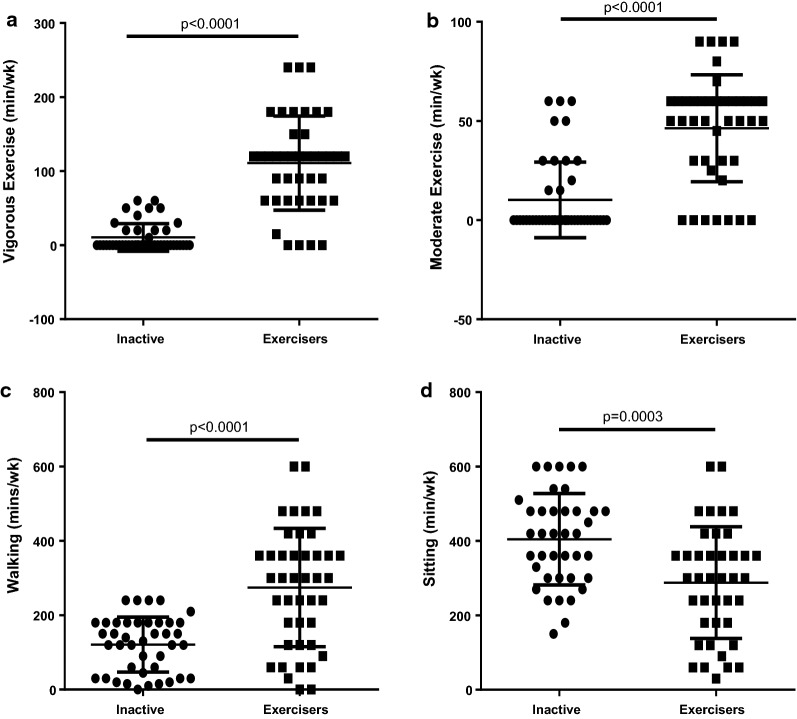
Exercise intensities in study population. **a** Vigorous exercise intensity, **b** moderate exercise intensity, **c** walking, **d** sitting duration. Variables are presented as mean ± standard deviation

**Fig. 2:**
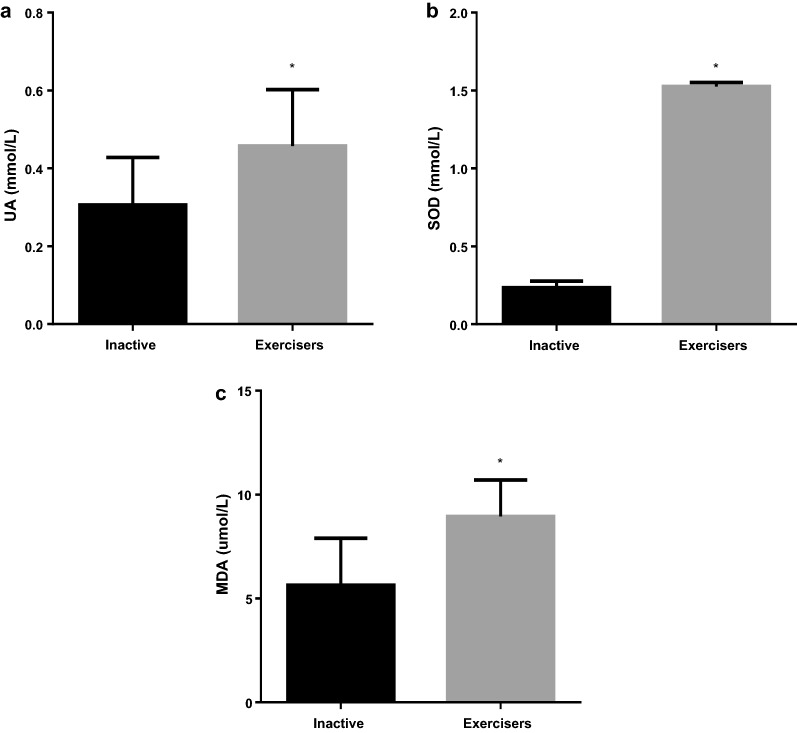
Antioxidants and oxidative stress parameters in study population. **a** *UA* uric acid, **b** *SOD* superoxide dismutase, **c** *MDA* malondialdehyde. Variables are presented as mean ± standard deviation. *p < 0.001 (vs inactive)

The biometric and biochemical data of the study population are shown in Table 1. Differences in age, height, BMI and heart rate between the two groups were not significant (p = 0.342, 0.476, 0.105 and 0.650 respectively). The exercisers exhibited significantly higher systolic blood pressure (SBP), diastolic blood pressure (DBP), muscle mass, daily calorie intake (DCI), metabolic age, bone mass and significantly lower total body weight (TBW), physic rating and visceral fat (p < 0.05) than their inactive counterparts. Concentrations of total cholesterol and low density lipoprotein cholesterol (LDL-C) were significantly increased in the inactive group compared with the exercisers, whereas high density lipoprotein cholesterol (HDL-C) concentrations were significantly increased in the exercise group (p < 0.05). As shown in Fig. 1, sub-groups performing different levels of intensity of exercise (vigorous, moderate, walking and decreased sitting time per week) within the exerciser group all exhibited significant increases of exercise compared with the inactive group (p < 0.001). In Fig. 2, concentration of the marker of oxidative stress (MDA) and concentration of antioxidants (SOD, UA) between the two groups were significantly different (p < 0.05). Gender difference among groups are shown in Additional file 4: Figure S1. Male exercisers exercised more vigorously compared to inactive males (p = 0.005) and inactive females (p < 0.001). They also participated in more moderate exercise than the inactive males (p = 0.028) and inactive females (p < 0.0001). The male exercisers had less sitting time/week compared to the inactive females (p < 0.0001). While the female exercisers participated in increased vigorous exercise compared to the inactive females (p < 0.045), they also had increased moderate exercise compared to inactive males (p = 0.0005) and inactive females (p < 0.001). Overall, males exercisers were more active than the inactive males (p = 0.002) but no change was observed with the female group. There was however, no significant difference among the male and female exercisers. The mean differences for TG, VLDL-C and fasting blood glucose (FBG) were not significant between the two groups. Associations of exercise intensities with UA, SOD and MDA are shown in Additional file 5: Table S1. There were positive correlations within the exercise group for vigorous exercise vs uric acid, SOD and MDA (r = 0.512, p = 0.004; r = 0.810, p = 0.001; r = 0.715, p = 0.001) and moderate exercise vs MDA (r = 0.841, p = 0.001) compared to the inactive group.

**Table 1:** Biometric and biochemical variables of study population

Variables	Inactive	Exercisers	p-value
Age	27.3 ± 7.8	29.1 ± 9.6	0.342
Sex (M/F)	9/35	32/10	–
Height (m)	1.6 ± 0.9	1.7 ± 0.1	0.476
Weight (kg)	67.3 ± 14.5	71.8 ± 14.3	0.151
BMI (kg/m^2^)	26.4 ± 4.3	24.7 ± 5.3	0.105
SBP (mmHg)	112.8 ± 4.3	119.7 ± 12.0	< *0.001*
DBP (mmHg)	71.3 ± 8.7	76.7 ± 11.2	*0.014*
Heart rate (bpm)	77.7 ± 12.2	76.7 ± 7.5	0.650
Body fat (%)	27.1 ± 10.1	24.1 ± 9.4	0.158
TBW (%)	62.8 ± 7.6	53.2 ± 6.2	< *0.001*
Muscle mass (kg)	43.6 ± 7.1	55.1 ± 8.4	< *0.001*
Physic rating	4.4 ± 1.5	3.7 ± 1.7	*0.046*
DCI (kcal/day)	2236.6 ± 494.0	3577.3 ± 628.7	< *0.001*
Metabolic age	27.2 ± 14.0	33.5 ± 13.1	*0.034*
Bone mass (kg)	2.3 ± 0.4	2.9 ± 0.5	< *0.001*
Visceral fat	6.2 ± 4.6	3.5 ± 2.4	< *0.001*
TC (mmol/L)	5.28 ± 1.04	4.39 ± 1.34	*0.001*
TG (mmol/L)	1.08 ± 0.43	0.94 ± 0.35	0.117
HDL-C (mmol/L)	1.60 ± 0.51	2.04 ± 0.28	< *0.001*
LDL-C (mmol/L)	2.74 ± 0.90	2.38 ± 1.09	*0.040*
VLDL-C (mmol/L)	0.45 ± 0.19	0.42 ± 0.16	0.549
FBG (mmol/L)	5.20 ± 1.80	4.95 ± 2.47	0.594

Variables are presented as mean ± standard deviation. Italic results indicate significant relationship

*BMI* body mass index, *SBP* systolic blood pressure, *DBP* diastolic blood pressure, *TBW* total body water, *DCI* daily calorie intake, *TC* total cholesterol, *TG* triglyceride, *HDL-C* high density lipoprotein cholesterol, *LDL-C* low density lipoprotein cholesterol, *VLDL-C* very low density lipoprotein cholesterol, *FBG* fasting blood glucose

### Discussion

The present study evaluated the effect of exercise on oxidative stress and selected metabolic parameters in young Ghanaian adults. Superoxide dismutase was elevated in the exercise group and correlated with vigorous exercise. A study that investigated the time course of exercise induction of SOD in human skeletal muscle found elevated levels of SOD after exercise [16]. Superoxide dismutase is a potent antioxidant acting as a detoxification enzyme converting oxygen radicals produced during intense exercise to hydrogen peroxide, a prooxidant that can cause detrimental effects to various cellular structures [17]. This study also revealed significant increases in UA among the exercise group. This possibly could be due to the high calories being used by the body for energy leading to the build-up of lactic acid that competes with uric acid for excretion during intense training, energy demand and dehydration [18]. In one study, serum UA increased with changes in LDL susceptibility due to oxidation during intense exercise [19, 20]. Uric acid is an abundant aqueous antioxidant that accounts for about two-thirds of all free radical-scavenging activity in human serum [21]. Its antioxidant ability enables it to scavenge carbon-centered and peroxyl radicals in hydrophilic states and may have an inhibitory effect on lipid peroxidation in lipid aqueous boundaries [22], preventing lipid peroxidation in skeletal muscle during high intensity exercise [23]. These findings suggest that the antioxidant properties of UA are of biological importance in vivo [24] and may arise from purine nucleotide degradation during conditions of high energy utilization [25].

Malondialdehyde, as a marker of oxidative damage, was accessed. The exact mechanism whereby MDA concentrations were increased after exercise was not investigated in this study, but exercise has previously been shown to increase oxidative and inflammatory damage [26, 27]. Exercise subjects performed more exercise than the inactive group, accounting in part to the increased MDA. Malondialdehyde is a product of autoxidation of unsaturated fatty acids. There are suggestions that mitochondria, nicotinamide adenine dinucleotide phosphate (NADPH) oxidase, phospholipase A_2_-dependent processes and xanthine oxidase [28, 29] may contribute to ROS production in humans, in turn leading to increased oxidative damage, a marker of which is MDA.

Healthy lifestyle changes may reduce the risk factors associated with diabetes and cardiovascular disease, with exercise enhancing the body’s ability to utilise the lipids in the body [3, 30]. Increased HDL-C and decreased TC concentrations were observed in the exercise group compared to the inactive group. Possibly, the mechanism of transfer of an ester group to HDL following increased levels of lecithin-cholesterol acyltransferase (LCAT) may have increased the HDL during exercise [31, 32]. An increase in HDL may also suggest a healthy cardiovascular system [33] though the gold standard in preventing cardiovascular risk is reduced TC levels [34] which was evident in this study. Both males and females performed more moderate to vigorous exercise than their inactive counterparts. A combined analysis for vigorous, moderate and walking intensity exercises showed exercised males were more active than their inactive male counterparts but no change was observed with the equivalent female groups. With the exception of Eastern European countries, men have previously been found to be physically more active than females and females reported more often the barriers to physical activity than men [10, 35]. These barriers may involve to some extent their involvement in multiple roles such as mother, wife, caregiver, employee, volunteer, desire to do other things and the failure to consider themselves as athletes which significantly affect their exercise time [36–38].

Being physically inactive has a severe public health consequence and is a major risk factor for NCDs [39–41]. An urgent need to make exercise a public health priority particularly in developing countries where the burden of NCDs is still a major challenge [14, 42], is warranted Hence concerted effort should be made to address the problems associated with longer sitting times, as both groups reported significant sitting time/week in this study. Inactivity arising from longer sitting time may be a major risk factor for the development of cardiovascular disease in Ghana [43]. This indeed has become more challenging with urbanisation and development leading to changes in modes of transport, with certain exercise becoming less. However, walking may generally be accepted as a usual form of exercise in Ghana, as the exercise group reported a significant amount of walking time, compared with the inactive group. As such, policymakers may find it relatively easy to adopt, develop and implement attractive programs to engage people in walking as a form of exercise as it is inexpensive and cost-effective in our socio-cultural setting.

The question of how much time or training the exercise should last remains debatable. The World Health Organisation (WHO) recommends 150 min of moderate-intensity exercise throughout the week [44] while 30–60 min of moderate-intensity exercise for 3–5 days of the week is recommended for adults by the Ministry of Health in Ghana [45]. It does suggest that while these recommendations may be useful as a guideline, specific population-based research should be tailored to individual settings. Exercise subjects in this study performed more exercise than what is recommended by the Ghana guidelines. This is a challenge, and the need for comprehensive representative data including the different population groups in Ghana with regards to exercise patterns is warranted. The Ministry of Health in Ghana could support and include social competitions and mass communication programs that seek to promote and achieve exercise-oriented goals at the community, regional and national levels. Indeed, these interventions can be enhanced through the involvement and engagement of non-governmental groups such as private, academics and sports organisation. Perhaps the creation and centralisation of affordable health promotion centers across the country for the public may be essential.

## Conclusion

The results of this study suggest that physical exercise increases antioxidant (superoxide dismutase and uric acid) concentrations, promotes health by increasing HDL-C concentration and reduces metabolic risk by decreasing total cholesterol levels. Walking could be an inexpensive yet beneficial way to encourage people to be physically active. However, exercise (vigorous) appears to increase oxidative stress. The implications of exercise intensity on oxidative stress and antioxidants merit further research.

## Limitations

There may be a potential bias in the self-reported participation of the physical activity questionnaire. Although the authors assessed the effect of physical activity status on some health biomarkers and antioxidant/oxidative stress biomarkers, a longitudinal study could be carried out in a future study. In addition, a further limitation was the inability to control the influence of diet on the key outcome variables.

## Additional files

**Additional file 1.** Questionnaire. Pretested interview questionnaire.

**Additional file 2.** Sample size determination and exercise programme. Sample size calculation and exercise programme.

**Additional file 3.** Anthropometric measurements and laboratory procedure. Body composition assessment and blood sampling.

**Additional file 4: Table S1.** Association between antioxidant concentration, oxidative stress and exercise intensity of study population.

**Additional file 5: Figure S1.** Gender difference of exercise intensities in study population. a) Vigorous exercise intensity b) Moderate exercise intensity c) Walking d) Sitting duration e) Combined exercise intensity (vigorous, moderate and walking) * p<0.05 (vs Inactive males); # p<0.05 (vs Inactive Females). Variables are presented as mean ± standard deviation.

## Abbreviations

ROS: reactive oxygen species
MDA: malondialdehyde
UA: uric acid
SOD: superoxide dismutase
NCDs: non-communicable diseases
BMI: body mass index
SBP: systolic blood pressure
DBP: diastolic blood pressure
TBW: total body water
DCI: daily calorie intake
FBG: fasting blood glucose
HDL: high density lipoprotein
LDL: low density lipoprotein
VLDL: very low density lipoprotein
TCHOL: total cholesterol
TG: triglyceride

## References

[1] Kushi LH, Doyle C, McCullough M, Rock CL, Demark-Wahnefried W, Bandera EV, Gapstur S, Patel AV, Andrews K, Gansler T (2012). American Cancer Society guidelines on nutrition and physical activity for cancer prevention. CA Cancer J Clin.

[2] Macera CA, Hootman JM, Sniezek JE (2003). Major public health benefits of physical activity. Arthritis Care Res.

[3] Pedersen BK, Saltin B (2006). Evidence for prescribing exercise as therapy in chronic disease. Scand J Med Sci Sports.

[4] Vina J, Sanchis-Gomar F, Martinez-Bello V, Gomez-Cabrera M (2012). Exercise acts as a drug; the pharmacological benefits of exercise. Br J Pharmacol.

[5] Warburton DE, Nicol CW, Bredin SS (2006). Health benefits of physical activity: the evidence. Can Med Assoc J.

[6] Young I, Woodside J (2001). Antioxidants in health and disease. J Clin Pathol.

[7] Seifried RM, Harrison E, Seifried HE, Boushey CJ, Coulston AM, Rock CL, Monsen E (2017). Antioxidants in health and disease. Nutrition in the prevention and treatment of disease.

[8] GSS (2010). 2010 Population and housing census: National Analytical Report.

[9] GSS (2015). Ghana demographic and health survey 2014.

[10] Guthold R, Ono T, Strong KL, Chatterji S, Morabia A (2008). Worldwide variability in physical inactivity: a 51-country survey. Am J Prev Med.

[11] Blair SN, LaMonte MJ, Nichaman MZ (2004). The evolution of physical activity recommendations: how much is enough?. Am J Clin Nutr.

[12] Asare M, Danquah SA (2015). The relationship between physical activity, sedentary behaviour and mental health in Ghanaian adolescents. Child Adolesc Psychiatry Mental Health.

[13] Abubakari A-R, Lauder W, Jones M, Kirk A, Agyemang C, Bhopal R (2009). Prevalence and time trends in diabetes and physical inactivity among adult West African populations: the epidemic has arrived. Public Health.

[14] WHO. Global status report on non-communicable disease. World Health Organisation; 2014. http://www.who.int/nmh/publications/ncd-status-report-2014/en/. Assessed 23 May 2017.

[15] Ainsworth BE, Haskell WL, Herrmann SD, Meckes N, Bassett DR, Tudor-Locke C, Greer JL, Vezina J, Whitt-Glover MC, Leon AS (2011). 2011 compendium of physical activities: a second update of codes and MET values. Med Sci Sports Exerc.

[16] Khassaf M, Child RB, McArdle A, Brodie DA, Esanu C, Jackson MJ (2001). Time course of responses of human skeletal muscle to oxidative stress induced by nondamaging exercise. J Appl Physiol.

[17] Giimiistas MK (2003). Lipid peroxidation, erythrocyte superoxide-dismutase activity and trace metals in young male footballers. Yonsei Med J.

[18] Palacios G, Pedrero-Chamizo R, Palacios N, Maroto-Sánchez B, Aznar S, González-Gross M (2015). Biomarkers of physical activity and exercise. Nutr Hosp.

[19] Benítez S, Sánchez-Quesada JL, Lucero L, Arcelus R, Ribas V, Jorba O, Castellví A, Alonso E, Blanco-Vaca F, Ordóñez-Llanos J (2002). Changes in low-density lipoprotein electronegativity and oxidizability after aerobic exercise are related to the increase in associated non-esterified fatty acids. Atherosclerosis.

[20] Huang L-L, Huang C-T, Chen M-L, Mao I-F (2010). Effects of profuse sweating induced by exercise on urinary uric acid excretion in a hot environment. Chin J Physiol.

[21] Maxwell S, Thomason H, Sandler D, Leguen C, Baxter M, Thorpe G, Jones A, Barnett A (1997). Antioxidant status in patients with uncomplicated insulin-dependent and non-insulin-dependent diabetes mellitus. Eur J Clin Invest.

[22] Muraoka S, Miura T (2003). Inhibition by uric acid of free radicals that damage biological molecules. Pharmacol Toxicol.

[23] Hellsten Y, Svensson M, Sjödin B, Smith S, Christensen A, Richter EA, Bangsbo J (2001). Allantoin formation and urate and glutathione exchange in human muscle during submaximal exercise. Free Radic Biol Med.

[24] Waring W, Convery A, Mishra V, Shenkin A, Webb D, Maxwell S (2003). Uric acid reduces exercise-induced oxidative stress in healthy adults. Clin Sci.

[25] Green HJ, Fraser IG (1988). Differential effects of exercise intensity on serum uric acid concentration. Med Sci Sports Exerc.

[26] Sallam N, Laher I (2016). Exercise modulates oxidative stress and inflammation in aging and cardiovascular diseases. Oxidative Med Cell Longev.

[27] Powers SK, Jackson MJ (2008). Exercise-induced oxidative stress: cellular mechanisms and impact on muscle force production. Physiol Rev.

[28] Powers SK, Nelson WB, Hudson MB (2011). Exercise-induced oxidative stress in humans: cause and consequences. Free Radic Biol Med.

[29] La Favor JD, Dubis GS, Yan H, White JD, Nelson MA, Anderson EJ, Hickner RC (2016). Microvascular endothelial dysfunction in sedentary, obese humans is mediated by NADPH oxidase: influence of exercise training. Arterioscler Thromb Vasc Biol.

[30] Earnest CP, Artero EG, Sui X, Lee D-c, Church TS, Blair SN. Maximal estimated cardiorespiratory fitness, cardiometabolic risk factors, and metabolic syndrome in the aerobics center longitudinal study. In: Mayo Clinic Proceedings: 2013. Elsevier; 2013. p. 259–70. 10.1016/j.mayocp.2012.11.006 PMC3622904 23391253

[31] Calabresi L, Franceschini G (2010). Lecithin: cholesterol acyltransferase, high-density lipoproteins, and atheroprotection in humans. Trends Cardiovasc Med.

[32] Riedl I, Yoshioka M, Nishida Y, Tobina T, Paradis R, Shono N, Tanaka H, St-Amand J (2010). Regulation of skeletal muscle transcriptome in elderly men after 6 weeks of endurance training at lactate threshold intensity. Exp Gerontol.

[33] Carroll MD, Kit BK, Lacher DA, Yoon S (2013). Total and high-density lipoprotein cholesterol in adults: National Health and Nutrition Examination Survey, 2011–2012. NCHS Data Brief.

[34] Whayne TF (2011). Atherosclerosis: current status of prevention and treatment. Int J Angiol.

[35] Sequeira S, Cruz C, Pinto D, Santos L, Marques A (2012). Prevalence of barriers for physical activity in adults according to gender and socioeconomic status. Br J Sports Med.

[36] Eyler AA, Vest JR, Sanderson B, Wilbur J, Matson-Koffman D, Evenson KR, Thompson JL, Wilcox S, Young DR (2002). Environmental, policy, and cultural factors related to physical activity in a diverse sample of women: the Women’s Cardiovascular Health Network Project—introduction and methodology. Women Health.

[37] Eyler AA (2003). Personal, social, and environmental correlates of physical activity in rural Midwestern white women. Am J Prev Med.

[38] Ainsworth BE, Wilcox S, Thompson WW, Richter DL, Henderson KA (2003). Personal, social, and physical environmental correlates of physical activity in African–American women in South Carolina. Am J Prev Med.

[39] Pratt M, Norris J, Lobelo F, Roux L, Wang G (2014). The cost of physical inactivity: moving into the 21st century. Br J Sports Med.

[40] Lee I-M, Shiroma EJ, Lobelo F, Puska P, Blair SN, Katzmarzyk PT, Group LPASW (2012). Effect of physical inactivity on major non-communicable diseases worldwide: an analysis of burden of disease and life expectancy. Lancet.

[41] Tenkorang EY, Kuuire V, Luginaah I, Banchani E (2017). Examining risk factors for hypertension in Ghana: evidence from the Study on Global Ageing and Adult Health. Glob Health Promot.

[42] Hallal PC, Bauman AE, Heath GW, Kohl HW, Lee I-M, Pratt M (2012). Physical activity: more of the same is not enough. Lancet.

[43] Addo PN, Nyarko KM, Sackey SO, Akweongo P, Sarfo B (2015). Prevalence of obesity and overweight and associated factors among financial institution workers in Accra Metropolis, Ghana: a cross sectional study. BMC Res Notes.

[44] Organization WH (2010). Global recommendations on physical activity for health.

[45] MoH (2010). Dietary and physical activity guidelines for Ghana.

