# Supplementary material for: Impact of exercise intensity on oxidative stress and selected metabolic markers in young adults in Ghana
Source: BMC Res Notes. 2018 Sep 3;11:634. doi: 10.1186/s13104-018-3758-y (PMC6126417; doi:10.1186/s13104-018-3758-y)
Supplement: Supplementary file 1 — Additional file 1. Questionnaire. Pretested interview questionnaire. [file 13104_2018_3758_MOESM1_ESM.docx]

**Questionnaire**

Name (in blocks)………………………………………………………………

Date ……………………………..…… Subject code…………………………………

Contact Tel. Number(s)……………………………………………………………..….

E-mail ……………………………………………………………………………..…....

Postal Address…………………………………………………………………….……

**DEMOGRAPHICS AND ANTHROPOMETRICS**

1. Age…………………………... 4. Weight (kg)………………………..
2. Height (m)…………………… 5. BMI………………………………….
3. BP…………………………… 6. Pulse………………………………….

7. % Body Fat……………… 8. % Total Body Water……………….

1. Muscle Mass…………… 10. Physique Rating……………….
2. DCI (Daily Calorie Intake)….……… 12. Metabolic Age……………
3. Bone Mass………………… 14.Viceral Fat……………

**Please tick [ ] the appropriate box where applicable**

15. Sex : [ ] Male [ ] Female

16. Marital status: [ ] Single [ ] Married

[ ] Divorced [ ] Widowed

17. Level of education [ ] None [ ] Primary

[ ] Secondary [ ] Tertiary

18. Occupation [ ] Unemployed

[ ] Trader/Self employed

[ ] Government worker

[ ] Others (pls. specify):……………………………

19. Ethnicity [ ] Akan

[ ] Ga

[ ] Ewe

[ ] Northerner

[ ] Others (pls. specify):…………………………….

**MEDICAL HISTORY**

1. Do you have any of the following conditions?

[ ] Chronic Kidney Disease

[ ] Hypertension

[ ] Diabetes Mellitus

[ ] Others (please specify

[ ] None

**HERBAL REMEDIES AND OTHER HEALTH PRODUCTS**

1. Are you taking any herbal remedy or preparation? [ ] Yes [ ] No
2. Are you taking any vitamins or antioxidant therapy? [ ] Yes [ ] No

**INTERNATIONAL PHYSICAL ACTIVITY QUESTIONNAIRE**

I am interested in finding out about the kinds of physical activities that you do as part of your everyday live. The questions will ask you about the time you spent being physically active in the **last 7 days**. Please answer each question even if you do not consider yourself to be an active person. Please think about the activities you do at work, as part of your house and yard work, to get from place to place, and in your spare time for recreation, exercise or sport.

Think about all the **vigorous** activities that you did in the **last 7 days**. **Vigorous** physical activities refer to activities that take hard physical effort and make you breathe much harder than normal. Think *only* about those physical activities that you did for at least 10 minutes at a time.

1. During the **last 7 days**, on how many days did you do **vigorous** physical activities like heavy lifting, digging, aerobics, or fast bicycling?

_____ **days per week**

No vigorous physical activities ***Skip to question 3***

1. How much time did you usually spend doing **vigorous** physical activities on one of those days?

_____ **hours per day**

_____ **minutes per day**

Don’t know/Not sure

Think about all the **moderate** activities that you did in the **last 7 days**. **Moderate** activities refer to activities that take moderate physical effort and make you breathe somewhat harder than normal. Think only about those physical activities that you did for at least 10 minutes at a time.

1. During the **last 7 days**, on how many days did you do **moderate** physical activities like carrying light loads, bicycling at a regular pace, or doubles tennis? Do not include walking.

_____ **days per week**

No moderate physical activities ***Skip to question 5***

1. How much time did you usually spend doing **moderate** physical activities on one of those days?

_____ **hours per day**

_____ **minutes per day**

Don’t know/Not sure

Think about the time you spent **walking** in the **last 7 days**. This includes at work and at home, walking to travel from place to place, and any other walking that you have done solely for recreation, sport, exercise, or leisure.

5. During the **last 7 days**, on how many days did you **walk** for at least 10 minutes at a time?

_____ **days per week**

No walking ***Skip to question 7***

1. How much time did you usually spend **walking** on one of those days?

_____ **hours per day**

_____ **minutes per day**

Don’t know/Not sure

The last question is about the time you spent **sitting** on weekdays during the **last 7 days**. Include time spent at work, at home, while doing course work and during leisure time. This may include time spent sitting at a desk, visiting friends, reading, or sitting or lying down to watch television.

1. During the **last 7 days**, how much time did you spend **sitting** on a **week day**?

_____ **hours per day**

_____ **minutes per day**

Don’t know/Not sure

**This is the end of the questionnaire, thank you for participating.**
