# Supplementary material for: Impact of exercise intensity on oxidative stress and selected metabolic markers in young adults in Ghana
Source: BMC Res Notes. 2018 Sep 3;11:634. doi: 10.1186/s13104-018-3758-y (PMC6126417; doi:10.1186/s13104-018-3758-y)
Supplement: Supplementary file 2 — Additional file 2. Sample size determination and exercise programme. Sample size calculation and exercise programme. [file 13104_2018_3758_MOESM2_ESM.docx]

**Title of data: Sample size determination and exercise programme**

**Description of data:** Sample size was calculated based on an absolute precision of 5%, a confidence interval of 95% and a non-response rate of 20%.

*Exercise programme*

Exercise intensity and duration was quantified as minutes/week using the International Physical Activity Questionnaire (IPAQ, 2002) (Short, self-administered format) [1]. The IPAQ consisted of 4 set questions that determined the participants average amount of vigorous and moderate exercise, as well as the amount of walking and sitting carried out each day. Vigorous exercise was classified as intensities similar to jogging, heavy lifting, digging, aerobics, or fast cycling. Moderate exercise was classified as exercise intensities equivalent to carrying light loads, cycling at a regular pace, or doubles tennis. The walking category included walking at work, at home, travelling from place to place, recreation, sports or leisure. Sitting included sitting at a desk or table, reading, and watching television.

**Reference**

1. Booth M: **Assessment of physical activity: an international perspective**. *Research Quarterly for Exercise & Sport* 2000, **71**(2):114-120.
