# Supplementary material for: Impact of exercise intensity on oxidative stress and selected metabolic markers in young adults in Ghana
Source: BMC Res Notes. 2018 Sep 3;11:634. doi: 10.1186/s13104-018-3758-y (PMC6126417; doi:10.1186/s13104-018-3758-y)
Supplement: Supplementary file 3 — Additional file 3. Anthropometric measurements and laboratory procedure. Body composition assessment and blood sampling. [file 13104_2018_3758_MOESM3_ESM.docx]

**Title of data: Anthropometric measurements and laboratory procedure**

**Description of data**: A body composition monitor (BC-533, Tanita innerscan^TM^ United Kingdom) which employed bio-impedance analysis to compute percentage body fat, body water percentage, muscle mass, bone mass, predicted daily calorie intake, metabolic age, total body water, physic rating and visceral fat was also used. Classification of BMI into categories was based on the World Health Organization criteria for adults [1]. Blood pressure (systolic and diastolic) was measured using an Omron digital 705 CP oscillometric monitor (Kyoto, Japan) after participants had rested for 15 min after exercise had stopped. The mean of 2 measurements was used as the final reading. Four millilitres of whole blood were drawn into a gel separator tube for processing. The resulting serum was separated into 0.5 mL aliquots in sterile Eppendorf tubes for lipid profile analyses as well as oxidative stress and antioxidant analysis. Serum samples were stored at -20^°^C until required. The remaining 1mL of blood was transferred into a fluoride-oxalate tube for fasting blood glucose concentration determination. The SOD and MDA concentrations were determined using spectrophotometric Bioassay systems assays. Fasting glucose, lipids and uric acid concentrations were analysed using auto-analysers (Biotecnica BT-3000 Plus, Rome, Italy).

**Reference**

1. WHO J, Consultation FE: **Diet, nutrition and the prevention of chronic diseases**. *World Health Organization Technical Report Series* 2003, **916**(i-viii).
