# Supplementary material for: Impact of exercise intensity on oxidative stress and selected metabolic markers in young adults in Ghana
Source: BMC Res Notes. 2018 Sep 3;11:634. doi: 10.1186/s13104-018-3758-y (PMC6126417; doi:10.1186/s13104-018-3758-y)
Supplement: Supplementary file 5 — Additional file 5: Figure S1. Gender difference of exercise intensities in study population. a) Vigorous exercise intensity b) Moderate exercise intensity c) Walking d) Sitting duration e) Combined exercise intensity (vigorous, moderate and walking) * p<0.05 (vs Inactive males); # p<0.05 (vs Inactive Females). Variables are presented as mean ± standard deviation. [file 13104_2018_3758_MOESM5_ESM.docx]

**Table S1 Association between antioxidant concentration, oxidative stress and exercise intensity of study population**

| Exercise Intensity | |  | Inactive |  |  | Exercisers |  |
| --- | --- | --- | --- | --- | --- | --- | --- |
|  | | UA | SOD | MDA | UA | SOD | MDA |
| Vigorous | r  p | 0.193  0.222 | 0.039  0.840 | -0.008  0.967 | *0.512*  *0.004* | *0.810*  *0.001* | *0.715*  *0.001* |
| Moderate | r  p | 0.080  0.605 | -0.139  0.473 | -0.090  0.641 | -0.083  0.606 | 0.180  0.341 | *0.841*  *0.001* |
| Walking | r  p | 0.135  0.401 | 0.214  0.283 | 0.134  0.504 | *0.455*  *0.012* | -0.085  0.655 | -0.065  0.733 |
| Sitting | r  p | *0.382*  *0.037* | -0.088  0.683 | *0.403*  *0.023* | -0.179  0.269 | -0.001  0.999 | -0.176  0.361 |

*Variables are presented as mean ± standard deviation. Italic results indicate significant relationship UA; uric acid, SOD; superoxide dismutase, MDA; malondialdehyde.*
