# Supplementary material for: Impact of exercise intensity on oxidative stress and selected metabolic markers in young adults in Ghana
Source: BMC Res Notes. 2018 Sep 3;11:634. doi: 10.1186/s13104-018-3758-y (PMC6126417; doi:10.1186/s13104-018-3758-y)
Supplement: Supplementary file 5 [file 13104_2018_3758_MOESM4_ESM.docx]

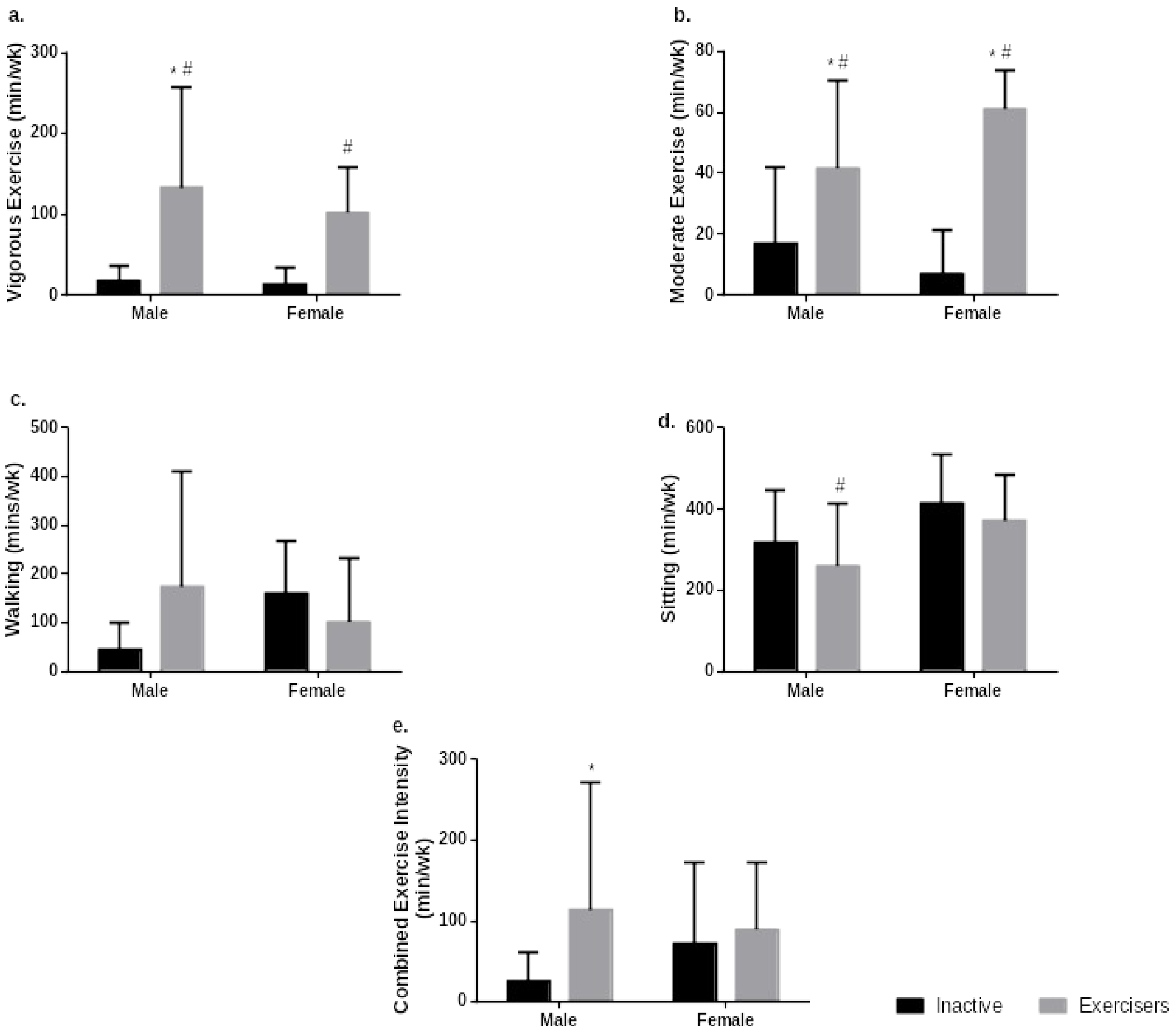
a.
Vigorous Exercise (min/wk)
0
100
200
300
* #
#
Male
Female
b.
Moderate Exercise (min/wk)
0
20
40
60
80
* #
* #
Male
Female
c.
Walking (mins/wk)
0
100
200
300
400
500
Male
Female
d.
Sitting (min/wk)
0
200
400
600
#
Male
Female
e.
Combined Exercise Intensity (min/wk)
0
100
200
300
*
Male
Female
Inactive
Exercisers
